# A coupled recreational anglers’ decision and fish population dynamics model

**DOI:** 10.1371/journal.pone.0206537

**Published:** 2018-10-31

**Authors:** Masami Fujiwara, Jesse D. Backstrom, Richard T. Woodward

**Affiliations:** 1 Department of Wildlife and Fisheries Sciences, Texas A&M University, College Station, TX, United States of America; 2 Department of Agricultural Economics, Texas A&M University, College Station, TX, United States of America; Fred Hutchinson Cancer Research Center, UNITED STATES

## Abstract

The effective management of fish populations requires understanding of both the biology of the species being managed and the behavior of the humans who harvest those species. For many marine fisheries, recreational harvests represent a significant portion of the total fishing mortality. For such fisheries, therefore, a model that captures the dynamics of angler choices and the fish population would be a valuable tool for fisheries management. In this study, we provide such a model, focusing on red drum and spotted seatrout, which are the two of the main recreational fishing targets in the Gulf of Mexico. The biological models are in the form of vector autoregressive models. The anglers’ decision model takes the discrete choice approach, in which anglers first decide whether to go fishing and then determine the location to fish based on the distance and expected catch of two species of fish if they decide to go fishing. The coupled model predicts that, under the level of fluctuation in the abundance of the two species experienced in the past 35 years, the number of trips that might be taken by anglers fluctuates moderately. This fluctuation is magnified as the cost of travel decreases because the anglers can travel long distance to seek better fishing conditions. On the other hand, as the cost of travel increases, their preference to fish in nearby areas increases regardless of the expected catch in other locations and variation in the trips taken declines. The model demonstrates the importance of incorporating anglers’ decision processes in understanding the changes in a fishing effort level. Although the model in this study still has a room for further improvement, it can be used for more effective management of fish and potentially other populations.

## Introduction

The effective management of fish stocks is achieved by understanding the behavior of anglers and creating appropriate incentives for sustainability [1]. In commercial fisheries, modern regulatory tools such as catch share programs [2] create such incentives. For example, the implementation of individual transferable quota (ITQ) in several major fisheries has reduced the fishing capacity while increasing the revenue and prolonging fishing seasons in the U.S. [3]. In comparison, it is often difficult to predict the behavior of anglers in recreational fisheries because multiple factors influence their behavior. Consequently, fishery managers are often forced to make management decisions without a good understanding of their effects on the behavior of recreational anglers [4].

Understanding the behavior of marine recreational anglers is important because recreational fishing effort is currently a significant portion of the total fishing effort in some parts of the ocean [5, 6] and understanding anglers’ behavior is critical to developing effective management. Among the species of concern in the northern Gulf of Mexico, it has been estimated that 64% of landing is taken by recreational anglers [6]. For example, in the federal water of the Gulf, the recreational quota of red snapper (*Lutjanus campechanus*) is currently almost equivalent to the commercial quota [7], and red drum (*Sciaenops ocellatus*) are caught exclusively by recreational anglers along the Texas coast. Therefore, consideration of marine recreational fishing activities is critically important for effective fishery management.

Recreational anglers make a series of behavioral decisions including whether to go fishing and where to go fishing. These decisions can ultimately influence the sustainability of fish stocks by affecting fish mortality. In the economics literature, these decisions are often modeled by assuming that anglers maximize their utility, which is affected by various factors such as the distance to fishing sites, expectation of catching specific species, and the availability of amenities at a fishing site [8]. Studying how these factors influence anglers’ decisions is an active area of research, but there have been few studies that have coupled fish dynamics and recreational anglers’ decision processes [9, 10].

**Fig 1:**
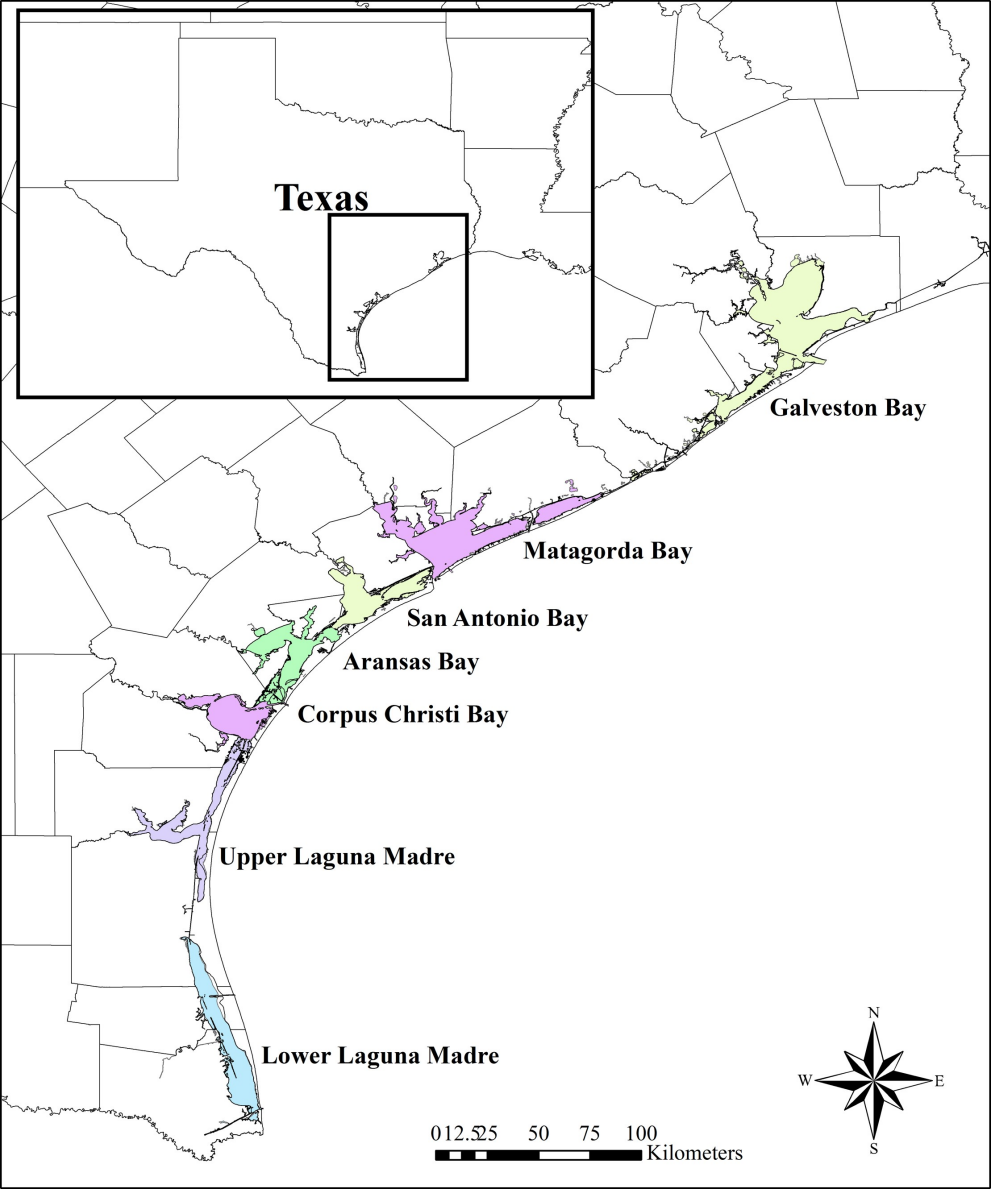
Map of Texas showing sampling locations (Bays).

Here, we develop a model that links fish population dynamics to anglers’ behavior. The objectives of this study are twofold. First, we demonstrate the potential utility of the model by parameterizing it from actual data. Second, we evaluate the idea that the anglers’ expectation of catching fish affects anglers’ decision to go fishing as well as the choice of locations they fish. The fish dynamic models are parameterized for red drum and spotted seatrout (*Cynoscion nebulosus*) using monitoring data available from seven major bays along the Texas coast (Fig 1). The anglers’ decision model was parameterized with data from the Marine Recreational Information Program (MRIP) [11] for recreational trips in west Florida, Alabama, and Mississippi. These locations were determined by the availability of the data. While the MRIP data are not ideal for estimating the behavior of Texas anglers, they are the best available data for estimating how the Gulf anglers’ choices are affected by the catch rates of the red drum and spotted seatrout. The portion of the MRIP data used in our analysis consists of 10,621 angler trips (5,425 with spotted seatrout reported as the primary target and 5,196 with red drum reported as the primary target) at 488 different sites in these three states, providing a rich empirical foundation for our analysis. We ssume that Texas anglers behave in a manner similar to anglers in these other Gulf states, which allows us to develop a model in which the biological and human behavioral processes are coupled.

In the MRIP data set, red drum and spotted seatrout are two of the most targeted species along the northern coast of the Gulf of Mexico by marine recreational anglers. As these two species are often potential alternative target species (in our data set, 2,870 trips reported red drum and spotted seatrout as primary and secondary target species or vice versa) it is desirable to develop a multi-species management strategy for them. As relative availability of the two species changes, anglers may shift their target from one species to the other. Alternatively, their decision may be based solely on the availability of one of the species. If the latter is the case, the capture of the other species may be viewed as bycatch.

Both red drum and spotted seatrout are considered estuarine fishes, but their life history and habitat use are different. The differences in life history and habitat use can produce differences in population dynamics between the two species. Red drum larvae hatch in fall months [12]. Once they hatch, larvae settle in marsh edge [13]. During juvenile stage, they mostly use estuaries, and as they reach maturity, they move to nearshore water [14]. During these stages, they are thought to exhibit site fidelity [15]. Adults make a long distance migration [16], and a majority of adults is thought to spawn in open nearshore water [14]. However, they are shown to have a homing behavior, returning closer to their estuarine nursery area [17]. On the other hand, spotted seatrout generally spawn from March to September in Texas although the season can vary geographically and annually [18]. During juvenile stages, they are found in seagrass bed [19], and all developmental stages of spotted seatrout are found in estuaries. Spotted seatrout are often considered non-migratory [20].

Our angler decision model takes the discrete choice approach, in which, anglers first decide whether to go fishing or not. Then, if they decide to go fishing, they choose the location (the bay) to go fishing based on the distance and expected catch. The biological models are in the form of vector autoregressive models [21], which are autoregressive models with interactions among multiple time series. In our study, fish species in multiple bays are thought to be associated with each other. These two models are linked by incorporating the predicted abundance of two species from the biological model as the expected catch in the angler’s decision model. This allows us to simulate the fluctuations in fish abundance and anglers’ decisions. The current model does not incorporate the feedback of anglers’ decisions to change in fish mortality, but we will discuss how it can be incorporated in the model in the discussion section, as it is an obvious and important extension of the model.

## Methods

### Simulating catch of red drum and spotted seatrout

In the biological models, we simulate the catch per unit effort (CPUE) of red drum and spotted seatrout in seven bays along the Texas Coast (Fig 1). The data have been collected twice a year (spring and fall) since 1982 by the Texas Parks and Wildlife Department [22]. These data are also described in Fujiwara et al. [23]. A total of 45 gillnets were set in each bay per season. The gillnets covered the water column from the seafloor to 1.2 meters above the bottom, had a total length of 182.9 m, and were constructed of four continuous 45.7 m-long panels of stretched mesh monofilament webbing of 152 mm, 127 mm, 102 mm, and 76 mm in size. Our analysis includes the data collected from 1982 to 2016, and we calculated the monthly CPUEs (the number of fish caught per hour) for each species in each bay. These data are available as Supporting Information (S1 Table). Because the monitoring data reflect the abundance of fish in the bays, we assume that the catch rates of recreational anglers vary proportionally with the monitoring data.

Our biological models take the form of vector autoregressive models (VARMs). Although VARMs are statistical simulation models, their structure can be constructed based on biological expectations. Here, we assume that the abundance of fish in a given bay is affected by the abundance of the same species in the same bay during the previous one year (i.e. two previous seasons) and that the environmental conditions affecting fish are correlated among neighboring bays. In addition, significant sampling error is expected in the monitoring data. Hence, our models take the form of second order VARMs, and are fitted to the data using a state-space method, which includes equations for both sampling and underlying biological processes [21].

The biological models take the following form:

$$x_{t,j}=b_{1,s}x_{t-0.5,j}+b_{2,s}x_{t-1,j}+w_{1,s}\varepsilon_{t,j-2}+w_{2,s}\varepsilon_{t,j-1}+w_{3,s}\varepsilon_{t,j}+w_{4,s}\varepsilon_{t,j+1}+w_{5,s}\varepsilon_{t,j+2}$$ (1)

where *x*_*t*,*j*_ is the state variable representing a measure of the abundance of fish at time *t* (*t* = 1,1.5,⋯,35.5) in location (bay) *j* (*j* = 1,⋯,7), *b*_*k*,*s*_and *w*_*k*,*s*_ are coefficients to be estimated, and *ε*_*t*,*j*_ is a process error with an independent normal distribution with mean 0 and variance 1. Index *s* denotes season: 1 for spring and 2 for fall. Time *t* is in years, but it is incremented by 0.5 from 1 to 35.5 because there are two sampling seasons (spring and fall) within a year. Index *k* specifies the location of parameter in the equation. The last two terms in the equation produce covariation in the process errors among neighboring bays. When neighboring bays do not exist because the bays are close to the edge, corresponding *w*_*k*,*s*_ was set to 0.

The above model assumes that there is temporal dependency on the two previous seasons. This assumption is based on the fact that majority of partial autocorrelation becomes insignificant beyond two previous seasons. The model also assumes association among three neighboring bays. This assumption is based on the fact the significant correlation of time series for each season is mostly within the three neighboring bays.

The observation equation is given as

$$y_{t,j}=x_{t,j}+v_{s}e_{t,j}$$ (2)

where *y*_*t*,*j*_ is the state variable reflecting the catch per unit effort, *v*_*s*_ is a coefficient to be estimated, and *e*_*t*,*j*_ is an observation error term, which takes an independent normal distribution with mean 0 and variance 1.

The full model consisted of 16 parameters. We assumed models always include the parameters associated with two observational error terms (*ν*_1_ and *ν*_2_) and two process error terms (*w*_4,1_ and *w*_4,2_). We constructed 4095 nested models by setting some of the other parameters to 0.

Fitting Eqs (1) and (2) requires that the data are stationary. Therefore, for each time series, we de-trended the original catch per unit effort (CPUE) data by fitting a polynomial equation of up to the third order with time as an independent variable. The order of the polynomial equation is selected using a leave-one-out cross validation method. Then, the residuals from the best model are added to the mean CPUE (the mean from 1982 to 2016 for each species in each bay) to obtain the de-trended time series. For de-trending, the data for each species for each bay in each season are analyzed separately, and we only deal with stationary part of the time series as extrapolating data using a polynomial function is problematic. The stationarity of de-trended time series is checked with the Augmented Dickey-Fuller (ADF) test using MATLAB function “adftest.m” with a time-lag of up to 3 and using AIC to select the best time-lag [24].

The de-trended data from spring and fall are combined for each species for each bay by lining them such that spring data is followed by fall data of the same year and fall data is followed by spring data of the following year. Then, combining the data for each species from the seven bays, we obtained two data sets of 70 x 7 (time × bays) observations: one for red drum and the other for spotted seatrout. The state variable *y*_*t*,*j*_ in Eq (2) denotes the de-trended CPUE at time *t* in location *j* after taking the natural log to stabilize the variance and then taking z-score of each column. For each of the two data sets, the VARMs are fitted using the function “estimate.m” in the Econometric Toolbox of MATLAB using the default setting [24], and the best model is selected using BIC. The estimated model is used for simulation using “simulate.m” in the Econometric Toolbox. Finally, after putting back the mean and variance used in the z-score to the simulated *y*_*t*,*j*_ and taking the exponential, we obtained simulated CPUEs, which is denoted by *c*_*t*,*j*_. Our model assumes the CPUE in spring will affect anglers during the following summer (high season), and that in fall will affect them during the following winter (low season).

### Simulating recreational demand for texas anglers

To simulate the behavior of recreational anglers, we apply the standard site-choice model used in recreation demand analysis [25]. In this model the probability of an angler, *i*, choosing a particular fishing location, *j* = 1, …,7 is a function of the expected catch rates at each of the possible locations *C* = (*c*1, …, *c*7) and the distance required by the angler to reach each of the 7 locations (bays), *D*_*i*_ = (*d*_*i*,1_,…,*d*_*i*,7_). The location choice is contingent on the angler’s decision to take a trip with the probability *P*_*T*_(*IV*) where *IV* refers to the *inclusive value* (to be defined below). We now explain the functional forms used in our simulation and explain how the parameters are estimated.

### Estimating the Probability of Choosing a Given Site

Following McFadden [26], we assume that the probability of taking a trip to location *j* is written as a logit function:

$$P_{j}\left(C,D\right)=\frac{e^{\beta_{d}d_{ij}+\beta_{c}c_{j}}}{\sum_{k=1}^{J}e^{\beta_{d}d_{ik}+\beta_{c}c_{k}}}$$ (3)

where βd and *βc* are the coefficients on distance and catch, respectively. These parameters were estimated using data from day-trip anglers from MRIP for 2013–2015, a three-year period after the effects of the Deep Water Horizon oil spill had dissipated.

MRIP, a program of the U.S. National Oceanic and Atmospheric Administration, conducts interviews with anglers at public access fishing sites through its Access Point Angler Intercept Survey. Surveyors are assigned to specific sites (i.e. 1–2 sites with similar characteristics) and specific times during the day. Interviews are carried out in six two-month waves throughout each year. Anglers are asked questions covering simple demographic information such as their home zip code; questions specific to their trip such as fishing mode, the primary and secondary targeted species, the number of people in their fishing party, the number of hours fished, number of days fished within the last year, and the number of fish caught by species; and questions specific to their catch for the day, including the number of fish (by species) that were caught, harvested, and released. Fish that are available at the time of the interview are measured and weighed. MRIP data are all publicly available from the program web page [11].

With thousands of site-intercept surveys conducted per year across the Gulf, MRIP collects the best data available for estimating recreational fishing behavior in the Gulf of Mexico. Unfortunately, Texas does not participate in MRIP and Louisiana halted its participation at the end of 2013. Hence, we model recreational behavior using data on fishing trips taken to sites in western Florida, Alabama, and Mississippi. To focus specifically on single-day fishing trips, we follow Lovell and Carter [27] and exclude anglers who traveled more than 150 miles (one-way) from their home zip code, and we reduce the size of their choice sets by excluding sites that are more than 150 miles away. Since our data do not include Texas, we do not include site-specific constants, which could not be obtained for the Texas bays in our simulation model. Hence, we assume that all variation in angler preferences is captured through changes in distance and the catch rates. The estimated coefficients on distance and the expected catch of red drum and spotted sea trout are presented in Table 1.

**Table 1:** Coefficient estimates for the angler’s responsiveness to distance, *β*_*d*_, and expected catch of red snapper and spotted sea trout, $\beta_{C}^{R}$ and $\beta_{C}^{S}$.

	*β*_*d*_	$\beta_{C}^{R}$	$\beta_{C}^{S}$
Coefficients	-0.082	0.170	0.347
Standard Errors	0.001	0.028	0.013

Because the recreation demand model is not estimated using Texas anglers, we do not have the number and distribution of the anglers throughout the state. Hence, our simulation model is calibrated assuming that there are *N* total trip occasions by representative anglers, all with identical distance options. Let ${\bar{c}}_{j}$ be the average expected catch rate at the *j*^th^ location and *Tj* be the average number of trips to that location. The CPUE values for the recreational anglers, ${\bar{c}}_{j}$, are found dividing the total harvests by the total trips for 2009–2010 using data from the Texas Parks and Wildlife Department (Table 2). Assuming that choices are made following the logit function written above, the share *T*_*j*_/∑*T*_*k*_ of a representative angler’s trips would be taken to site *j* if

$$\frac{T_{j}}{\sum T_{k}}=P_{j}\left(\bar{C},\bar{D}\right)=\frac{e^{\beta_{d}{\bar{d}}_{j}+\beta_{c}c_{j}}}{\sum_{k}e^{\beta_{d}{\bar{d}}_{k}+\beta_{c}c_{k}}}\;j=1,\ldots ,7$$ (4)

With knowledge of ${\bar{c}}_{j}$ for each of the bays, it is possible to solve the system of equations to find values for ${\bar{d}}_{j}$ consistent with the observed distribution of trips for all but one of the bays. For notational convenience, let $D_{j}=e^{\beta_{d}{\bar{d}}_{j}}$ and $C_{j}=e^{\beta_{c}{\bar{c}}_{j}}$ so, can be rewritten as $\frac{T_{j}}{\sum T_{k}}=\frac{D_{j}C_{j}}{\sum_{k}D_{k}C_{k}}$. We can then express *D*_2_ as a function of *D*_1_:

$$\frac{D_{2}C_{2}}{D_{1}C_{1}}=\frac{T_{2}}{T_{1}}\Rightarrow D_{2}=\frac{T_{2}}{T_{1}}\frac{C_{1}}{C_{2}}D_{1}$$ (5)

Hence, with values for ${\bar{d}}_{1}$, *β*_*c*_, *β*_*d*_, ${\bar{c}}_{1}$, ${\bar{c}}_{2}$, *T*_1_ and *T*_2_, we can solve for *D*2, *D*3, and so on, and then ${\bar{d}}_{2},\cdots ,{\bar{d}}_{7}$ by extension. After arbitrarily setting ${\bar{d}}_{1}=100$, the remaining distances ${\bar{d}}_{j}$ (for *j* = 2,⋯,7) are calculated using this approach, and those distances are shown in Table 3. Notice that the estimated distances are *not* representative of any actual point in Texas, but are instead a set of distances that would need to be faced by a hypothetical representative angler if the distribution of such an angler’s trips were to be the same as that observed in the Texas Parks and Wildlife data. Since catch rates and the trips are reported for both the high and low seasons, two different distance vectors are reported in Table 3.

**Table 2:** Base values for effort (private boat trips) and landings 2009–2010 season.

	Galveston (GL)	Matagorda (MG)	San Antonio (SA)	Aransas (AR)	Corpus Christi (CC)	Upper Laguna Madre (UL)	Lower Laguna Madre (LL)
	**Base Effort: Average number of trips per season by bay**						
High Season	191,165	75,973	57,864	92,666	62,542	83,636	68,104
Low Season	50,761	29,020	19,232	52,620	35,516	40,916	33,185
	**Base Harvest Average number of fish landed per season by bay**						
	(Red Drum)						
High Season	30,997	13,471	14,321	14,930	13,898	19,488	17,097
Low Season	7,243	5,575	5,035	8,461	5,655	4,143	6,050
	(Spotted Sea Trout)						
High Season	104,042	54,118	28,796	20,811	20,298	76,372	56,830
Low Season	17,865	10,406	12,892	16,045	6,746	18,537	15,154

Source: Data from the Texas Marine Sport-Harvest Monitoring Program, Personal Communication, Mark Fisher (Texas Parks and Wildlife Department)

**Table 3:** Distance vector for representative angler used to calibrate the model.

	Galveston	Matagorda	San Antonio	Aransas	Corpus Christi	Upper Laguna Madre	Lower Laguna Madre
High Season	100.0	112.0	114.6	107.5	112.8	111.8	114.0
Low Season	100.0	106.9	113.4	99.4	103.7	103.0	105.7

Because we have separate catch coefficients for red drum and spotted sea trout, in practice, $C_{j}=e^{\beta_{c}^{R}{\bar{c}}_{j}^{R}+\beta_{c}^{S}{\bar{c}}_{j}^{S}}$, where the superscripts *R* and *S* refer to red drum and spotted sea trout, respectively. Since, the distribution of the trips taken to the seven bays differs between the high (summer) and low (winter) seasons, we obtain two distance vectors $\bar{d}$, one for each season.

Anglers can not know exactly the catch that they will achieve when taking a trip. Instead they must base their choices on their expected catch at a site. We assume that an angler’s expectation of the catch at site *j* in period *t*, *E*(*c*_*jt*_), is a equal to a weighted average of catch rates as sites in the vicinity in both the current and previous year:

$$E\left(c_{j,t}\right)=\frac{\Sigma_{k}\omega^{dist}\left(k,j\right)\omega^{wv}\left(k,t\right)\omega^{yr}\left(k,t\right)\left(c_{k}-{\bar{c}}_{k}\right)}{\Sigma_{k}\omega^{dist}\left(k,j\right)\omega^{wv}\left(k,t\right)\omega^{yr}\left(k,t\right)}+{\bar{c}}_{j}$$ (6)

where ${\bar{c}}_{j}$ is the historical average catch at site *j*, *ω*^*dist*^ is the weight given to observed landings as distance from the site *j* increases, *ω*^*wv*^ is the weight for landings in previous waves in the same year, and *ω*^*yr*^ is for landings in the same wave in the previous year. The weights are estimated using the MRIP reported catch data for 2012–2015, assuming that on average anglers are able to estimate catch at a site using reports of catch at that and other sites. The distance weight, *ω*^*dist*^ is estimated to decline at the rate of 8% per mile for spotted sea trout and 1% per mile for red drum. Compared to the same period, the weight given to catch in the previous season, *ω*^*wv*^, was 0.35 for spotted sea trout and 0.54 for red drum. Finally, we find that harvests one year prior, *ω*^*yr*^, are given a weight of 0.84 for spotted sea trout and the previous year receives essentially no weight in calculating the expected catch for red drum. Because of this weighted formulation, the expected catch rates used in our simulation evolve more smoothly than actual catch rates over both time and space.

### Probability of taking a trip

The estimated coefficients for the recreation demand model are contingent on a trip being taken; they do not allow us to actually recover that an angler might not actually take a trip. Following Dundas et al. [28], we assume that the representative anglers’ decision to take a trip is a function of the inclusive value, $IV=\ln\left(\sum_{k}e^{\beta_{d}d_{k}+\beta_{c}c_{k}}\right)$, which can be thought of as an angler’s expected utility of a taking a trip [29]. Assuming the probability of taking a trip, *P*_*T*_, is a logistic function, it can be written

$$P_{T}^{}\left(IV\right)=\frac{\exp\left(\alpha +\lambda \cdot IV\right)}{1+\exp\left(\alpha +\lambda \cdot IV\right)}$$ (7)

where α is a set of other variables that affect the decision about whether to take a trip and λ is a coefficient that captures the trip-no trip decision, frequently referred to as the dissimilarity coefficient. A marginal increase in the *IV*, therefore, affects the probability that an angler will take a trip as follows:

$$\frac{\partial P_{T}}{\partial IV}=\frac{\lambda \exp\left(\alpha +\lambda \cdot IV\right)}{\left(1+\exp\left(\alpha +\lambda \cdot IV\right)\right)}-\frac{\lambda \exp\left(2\left(\alpha +\lambda \cdot IV\right)\right)}{{\left(1+\exp\left(\alpha +\lambda \cdot IV\right)\right)}^{2}}=\lambda \left[1-P_{T}\right]P_{T}$$ (8)

With knowledge of *λ* and *P*_*T*_, therefore, we can estimate the change in *P*_*T*_ associated with a change in *IV* using a linear approximation, $\Delta P_{T}=\frac{\partial P_{T}}{\partial IV}\Delta IV$, and *ΔIV* can be calculated by simply substituting in the base and simulated values of *C* and taking the difference.

A standard approach to estimating *λ* is to use a nested-logit model in which the first nest relates to the trip-no trip decision, and the second nest captures the site choice. Our data does not allow us to estimate the first nest, so we cannot directly estimate the value of *λ*. Instead, we follow the approach used by Dundas et al. [28] to infer the value of *λ* from an estimate of the value of a fishing trip. If *γ*^*TC*^ is the travel cost coefficient in a nested logit model, then the willingness to pay for a day of fishing (*WTP*) can be estimated using the equation, *WTP* = −1/*γ*^*TC*^ [30]. Dundas et al. [28] show that the travel cost coefficient, *γ*^*TC*^, is equal to *β*/*λ*, where *λ* is the dissimilarity coefficient in a nested logit model, and *β* is the coefficient on travel cost in the site-choice part of the model, i.e. conditional on taking a trip. If *WTP* is known, therefore, one can recover the associated value of *λ* using the equation *λ* = −*β*⋅*WTP*. There is a wide range of *WTP* estimates for saltwater recreational trips in the literature, ranging from less than a dollar, to several hundred dollars. Two recent meta-analysis studies [31, 32] find average WTP values for saltwater angling trips of $27.70 and $36.42 based on 14 and 5 studies respectively. Hence, we follow Dundas et al. [28] and use a *WTP* of $30 to calibrate our model. Since the estimated coefficient on our distance parameter is −0.0820, this suggests a value for *λ* of 2.46. In our analysis, the conditional logit model is estimated using travel distance rather than travel cost.

Our estimated coefficient, *β*_*d*_, is equal to the coefficient on travel cost only if travel cost is exactly proportional to travel distance and the cost per mile of round trip is exactly $1 (i.e. 50¢ per mile traveled). In our simulation analysis, we present the results assuming three possible values for the one-way mile cost per mile: $0.5 $1.0 and $2.0, each of which is associated with a different value of *λ*.

Eq (8) can be used to estimate changes in the probability of a trip, *P*_*T*_, from a given base level. We used the 2004 survey of Texas anglers to obtain base levels of participation [33]. That survey found that the average salt-water angler fished 20 days in the year, an annual probability of taking a trip of 20/365 = 5.48%. We will rescale this so that the average is spread out across the high-use and low-use seasons proportionally with 176 days in the low season and 189 days in the high season. In that case, $5.48\%=\frac{P_{Lo}\cdot 176+P_{Hi}\cdot 189}{365}$ so $P_{Hi}=5.48\%\cdot \frac{365}{189}\left(\frac{Trips_{Hi}}{Trips_{Lo}+Trips_{Hi}}\right)$ and $P_{Lo}=\frac{Trips_{Lo}}{Trips_{Hi}}P_{Hi}\cdot \frac{189}{176}$, yielding *PHi* = 0.0749 and *PLo* = 0.0332.

### Elasticities of trips with respect to catch

We also calculate how a change in the catch rate at a given bay affects the probability of fishing at that bay and, therefore, the predicted number of trips taken. The probability that an angler on a given day would take a trip to location *j* is $P_{j}^{T}=P_{T}P_{j}$. Let $\varepsilon_{j}^{T}$ be the elasticity of the percentage change in the $P_{j}^{T}$ for a change in *cj*, i.e. $\varepsilon_{j}^{T}=\frac{\partial P_{j}^{T}}{\partial c_{j}}\frac{c_{j}}{P_{j}^{T}}$, which can be simplified as the sum of two elasticities,

$$\frac{\partial P_{j}^{T}}{\partial c_{j}}\frac{c_{j}}{P_{j}^{T}}=\frac{\partial P_{T}}{\partial c_{j}}\frac{c_{j}}{P_{T}}+\frac{\partial P_{j}}{\partial c_{j}}\frac{c_{j}}{P_{j}}=\varepsilon_{T}^{c_{j}}+\varepsilon_{j}^{c}$$ (9)

where $\varepsilon_{T}^{c_{j}}$ is the elasticity of the probability of taking a trip, and $\varepsilon_{j}^{c}$ is the elasticity of the site-choice probability. Using (8), it follows that $\frac{\partial P_{T}}{\partial c_{j}}=\lambda \left[1-P_{T}\right]P_{T}\frac{\partial IV}{\partial c_{j}}$, and since $\frac{\partial IV}{\partial c_{j}}=\frac{\beta_{j}e^{\beta_{d}{\bar{d}}_{j}+\beta_{c}c_{j}}}{\sum_{k}e^{\beta_{d}{\bar{d}}_{k}+\beta_{c}c_{k}}}=\beta_{c}P_{j}$, we can write $\frac{\partial P_{T}}{\partial c_{j}}=\lambda \left[1-P_{T}\right]P_{T}\beta_{c}P_{j}$. Hence, the elasticity of the probability of taking a trip can be written,

$$\varepsilon_{T}^{c_{j}}=\lambda \left[1-P_{T}\right]P^{T}\beta_{c}P_{j}\frac{c_{j}}{P^{T}}=\lambda \left[1-P_{T}\right]\beta_{c}P_{j}c_{j}$$ (10)

The elasticity of the site-choice probability, $\varepsilon_{j}^{c}$, can be simplified since

$$\frac{\partial P_{j}}{\partial c_{j}}=\frac{\beta_{c}e^{\beta_{c}c_{j}+\beta_{d}d_{j}}}{e^{IV}}-\frac{\beta_{c}e^{2\left(\beta_{c}c_{j}+\beta_{d}d_{j}\right)}}{{\left(e^{IV}\right)}^{2}}=\beta_{c}\left(P_{j}-P_{j}^{2}\right)$$ (11)

so that

$$\varepsilon_{j}^{c}=\frac{\partial P_{j}}{\partial c_{j}}\frac{c_{j}}{P_{j}}=\beta_{c}\left(P_{j}-P_{j}^{2}\right)\frac{c_{j}}{P_{j}}=\beta_{c}c_{j}\left(1-P_{j}\right)$$ (12)

Hence, we can finally write the sum,

$$\varepsilon_{j}^{T}=\lambda \left[1-P_{T}\right]\beta_{c}P_{j}c_{j}+\beta_{c}c_{j}\left(1-P_{j}\right)$$ (13)

## Results

**Fig 2:**
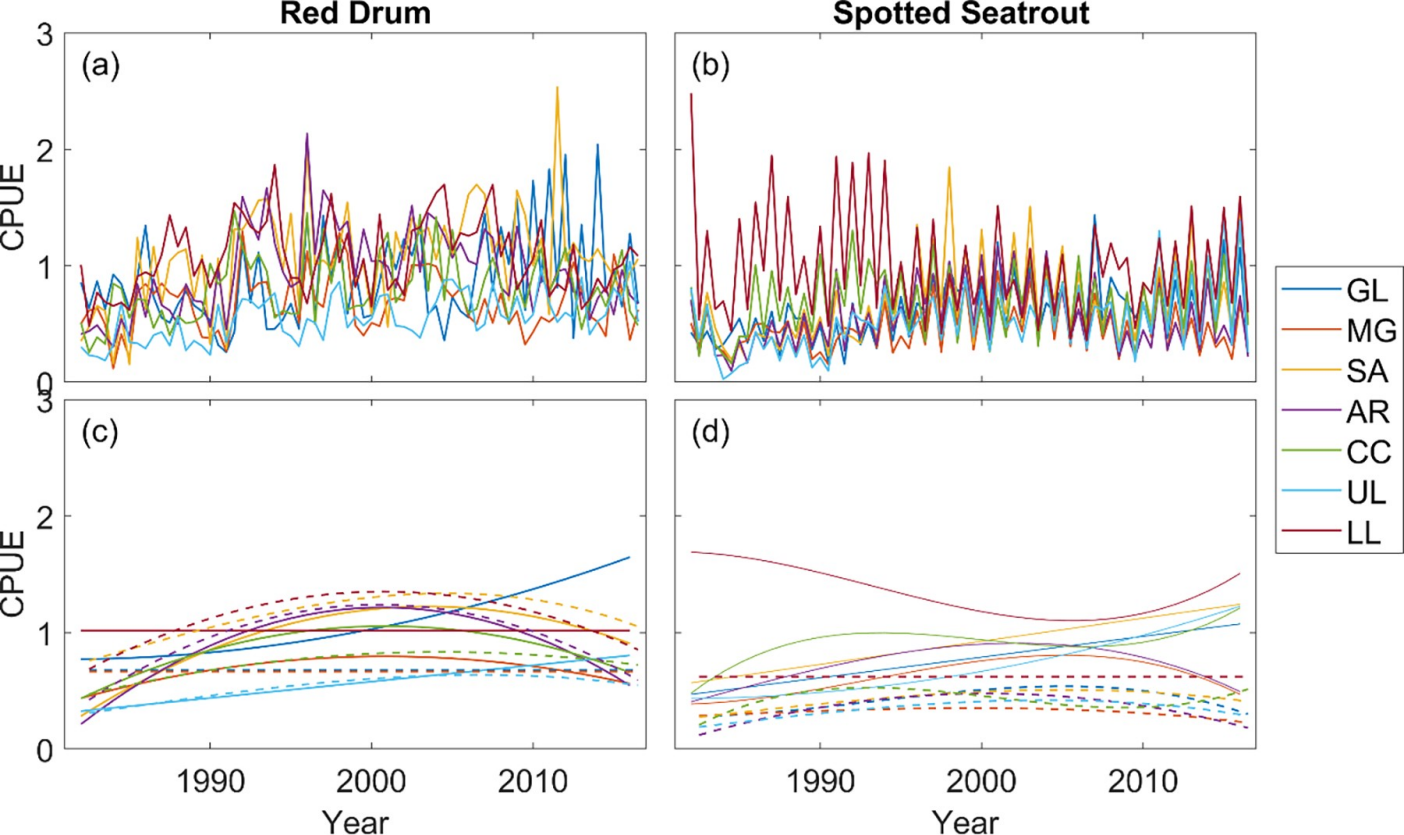
Catch per unit effort (CPUE) of red drum and spotted seatrout from 1982 to 2016. (a) & (b): the original CPUE. (c) & (d): fitted polynomials to the original data (solid lines: spring CPUE; dashed lines: fall CPUE). GL: Galveston Bay; MG: Matagorda Bay; SA: San Antonio Bay; AR: Aransas Bay; CC: Corpus Christi Bay; UL: Upper Laguna Madre; LL: Lower Laguna Madre.

**Fig 3:**
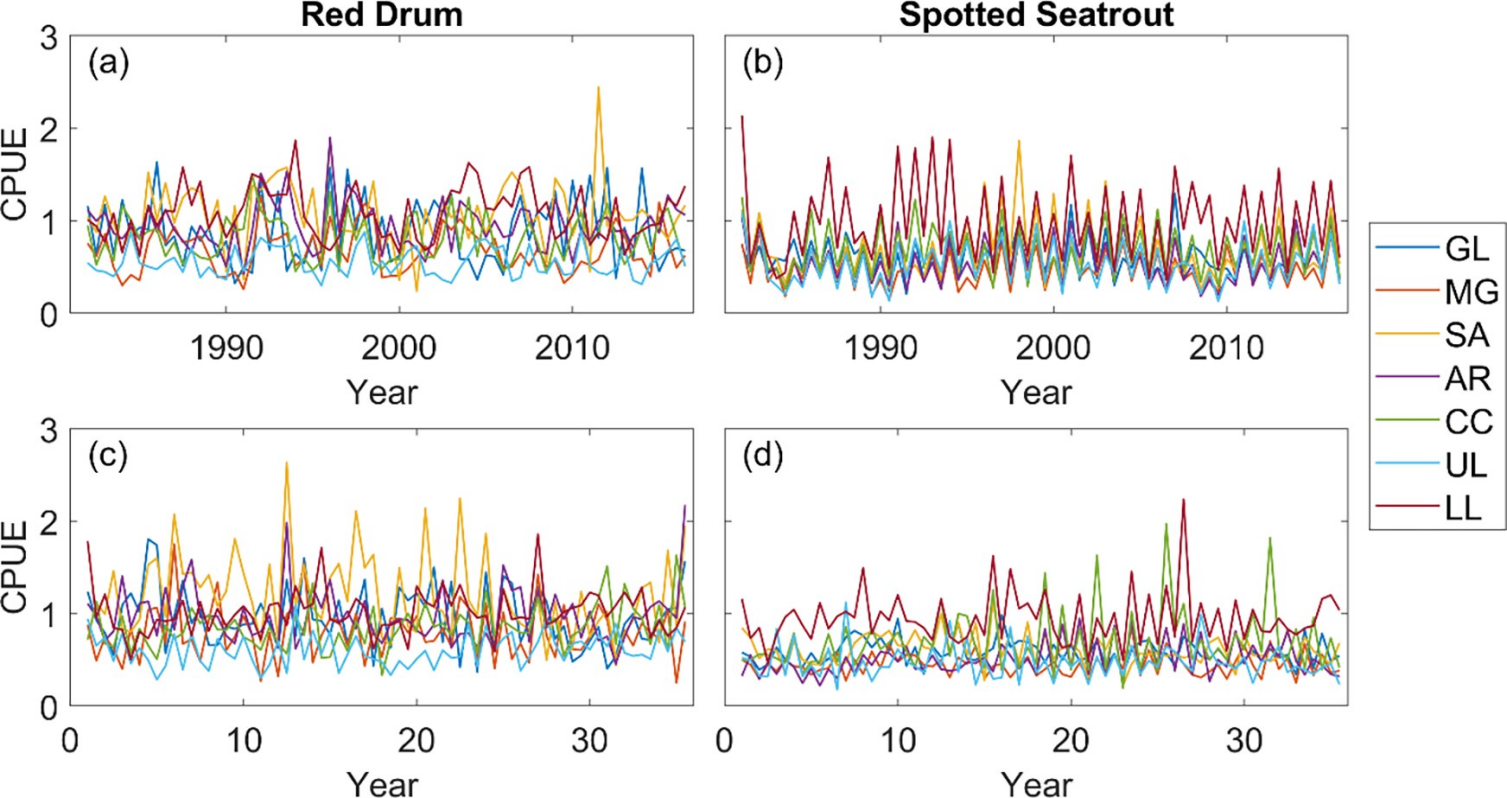
De-trended CPUE of red drum and spotted seatrout. (a) & (b) De-trended original time series, (c) & (d) Time series simulated over 35 years with the fitted VARM models. GL: Galveston Bay; MG: Matagorda Bay; SA: San Antonio Bay; AR: Aransas Bay; CC: Corpus Christi Bay; UL: Upper Laguna Madre; LL: Lower Laguna Madre.

We analyzed 28 fishery-independent time series of catch per unit effort (CPUE) (2 seasons x 7 bays x 2 species), and 23 of them were not stationary (Fig 2). After removing trends (see Methods), all the series became stationary (Fig 3A & 3B), which was confirmed with the ADF test (*α* = 0.1). Using the stationary time series, 4095 VARMs were fitted to the data for each species. The likelihood estimation was repeated 5 times with different initial conditions for each model, but parameters in some models were not estimable. We eliminated those models, and the best model among the remaining models was selected using BIC for red drum and spotted seatrout separately (S2 and S3 Tables). The best models are shown in Table 4. Using the best models, red drum and spotted seatrout catches were simulated over 35 years (Fig 3C & 3D).

**Table 4:** Estimated state-space model equations. Spring equations are for predicting the spring state variables, and fall equations are for predicting the fall state variables. Values in parentheses are estimated standard errors.

**Red Drum**			
Selected Model (Spring)	*x*_*t*,*j*_ = *b*_2,1_*x*_*t*−1,*j*_ + *w*_1,1_*ε*_*t*,*j*−2_ + *w*_3,1_*ε*_*t*,*j*_ + *w*_4,1_*ε*_*t*,*j*+1_		
	*y*_*t*,*j*_ = *x*_*t*,*j*_ + *v*_1_*e*_*t*,*j*_		
Estimated Parameters (Spring)	*b*_2,1_: 0.33 (0.11)	*w*_1,1_: 0.27 (0.13)	*w*_3,1_: 0.66 (0.21)
	*w*_4,1_: 0.42 (0.17)	*v*_1_: 0.66 (0.13)	
Selected Model (Fall)	*x*_*t*,*j*_ = *b*_2,2_*x*_*t*−1,*j*_ + *w*_1,2_*ε*_*t*,*j*−2_ + *w*_2,2_*ε*_*t*,*j*−1_ + *w*_3,2_*ε*_*t*,*j*_		
	*y*_*t*,*j*_ = *x*_*t*,*j*_ + *v*_2_*e*_*t*,*j*_		
Estimated Parameters (Fall)	*b*_2,2_: 0.23 (0.15)	*w*_1,2_: 0.18 (0.09)	*w*_2,2_: 0.28 (0.11)
	*w*_3,2_: 0.74 (0.25)	*v*_2_: 0.34 (0.46)	
**Spotted Sea Trout**			
Selected Model (Spring)	*x*_*t*,*j*_ = *b*_2,1_*x*_*t*−1,*j*_ + *w*_1,1_*ε*_*t*,*j*−2_ + *w*_2,1_*ε*_*t*,*j*−1_ + *w*_3,1_*ε*_*t*,*j*_ + *w*_4,1_*ε*_*t*,*j*+1_ + *w*_5,1_*ε*_*t*,*j*+2_		
	*y*_*t*,*j*_ = *x*_*t*,*j*_ + *v*_2_*e*_*t*,*j*_		
Estimated Parameters (Spring)	*b*_2,1_: 0.91 (0.03)	*w*_1,1_: 0.12 (—)	*w*_2,1_: 0.17(0.04)
	*w*_3,1_: 0.13 (0.03)	*w*_4,1_: 0.16 (0.04)	*w*_5,1_: 0.14 (0.03)
	*ν*_1_: 0.45 (0.03)		
Selected Model (Fall)	*x*_*t*,*j*_ = *b*_1,2_*x*_*t*−0.5,*j*_ + *b*_2,2_*x*_*t*−1,*j*_ + *w*_1,2_*ε*_*t*,*j*−2_ + *w*_2,2_*ε*_*t*,*j*−1_ + *w*_3,2_*ε*_*t*,*j*_ + *w*_4,2_*ε*_*t*,*j*+1_		
	*y*_*t*,*j*_ = *x*_*t*,*j*_ + *v*_*s*_*e*_*t*,*j*_		
Estimated Parameters (Fall)	*b*_2,1_: 0.61 (0.11)	*b*_2,2_: -0.29 (0.11)	*w*_1,2_: 0.21 (0.06)
	*w*_2,2_: 0.25 (0.0.07)	*w*_3,2_: 0.14 (0.05)	*w*_4,2_: 0.39 (0.06)
	*v*_2_: 0.41 (0.04)		

Note: For Galveston Bay (*j* = 1), *w*_1,*s*_ = 0 and *w*_2,*s*_ = 0. For Matagorda Bay (*j* = 2), *w*_1,*s*_ = 0. For Upper Laguna Madre (j = 8), *w*_5,*s*_ = 0. For Lower Laguna Madre (*j* = 7), *w*_4,*s*_ = 0 and *w*_5,*s*_ = 0.

**Fig 4:**
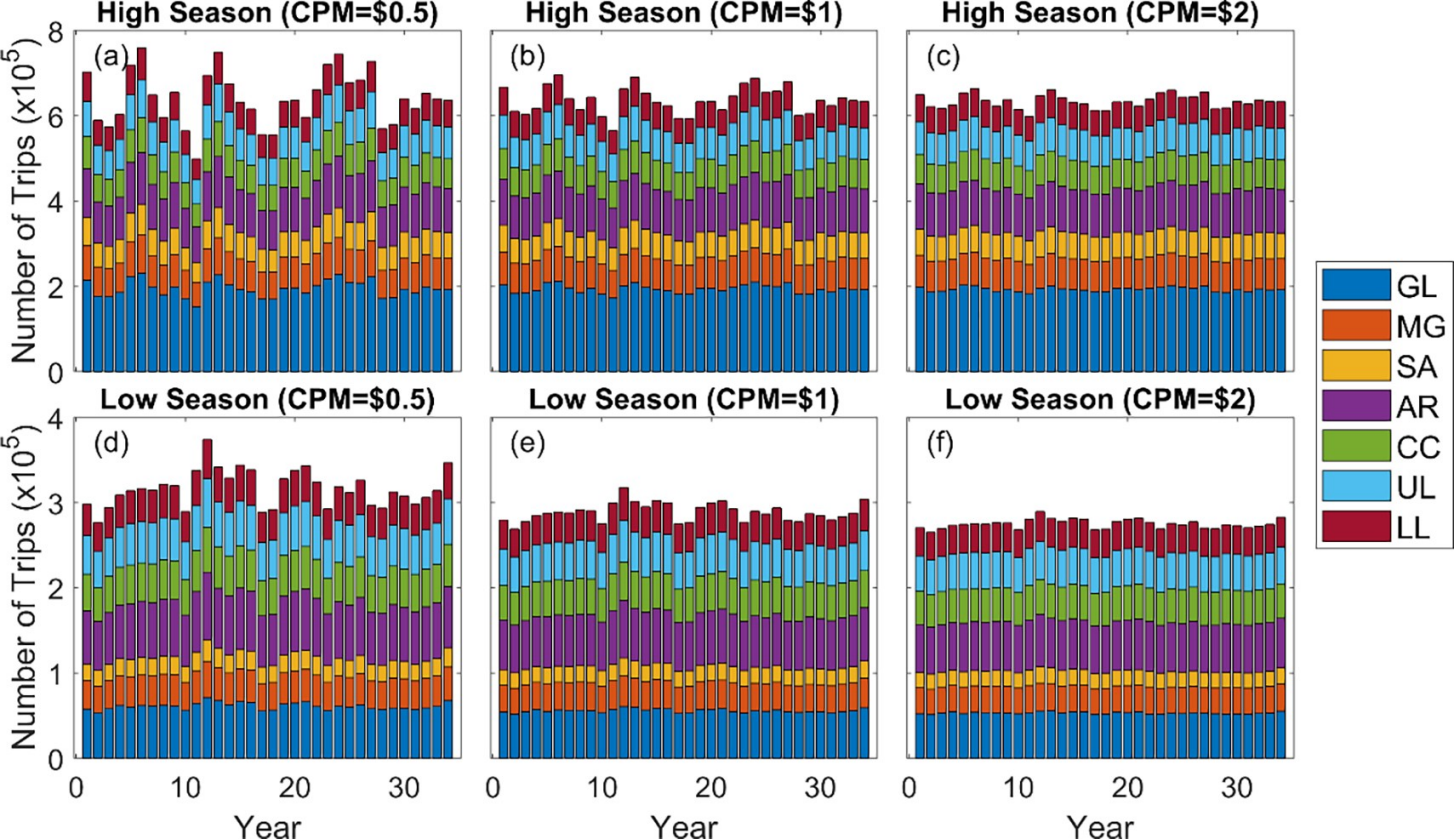
Simulated number of trips to the seven major bays. Trips during high season with the cost per mile (CPM) of $0.5 (a), $1 (b), and $2 (c), and trips during low season with the cost per mile (CPM) of $0.5, (d), $1 (e), and $2 (f). The variation comes from the variation in catch rate (see Fig 3C & 3D). GL: Galveston Bay, MG: Matagorda Bay, SA: San Antonio Bay, AR: Aransas Bay, CC: Corpus Christi Bay, UL: Upper Laguna Madre, LL: Lower Laguna Madre.

The simulated CPUEs were then used to create a series of expected catch values for the representative angler. Using the model described above, those values can be used to predict the number of trips taken to each of the 7 bays using the estimated site-choice model described in the method section. Fig 4 shows the simulation of the number of trips taken for each bay based on simulated catch shown in Fig 3C and 3D. The temporal fluctuation in the number of trips is moderate and caused by the temporal fluctuation in the catch rate. The model is evaluated for three different values of *λ*, associated with costs per one-way mile of $0.5, $1.0 and $2.0. As is seen in the figure, the amount of variation predicted is greater if the travel cost is lower.

Evaluating Eq (13) at the base levels of catch and trips, we obtained elasticity estimates in Table 5. We present the elasticities for the same three values of cost per mile and find, for example, that a 10% increase in the spring red drum catch rate in Galveston would result in an increase in the number of trips taken to that bay of between 1.2% and 2.3% during the high season as the cost per mile is varied from $0.5 to $2. The elasticities for spotted seatrout, on the other hand, are about three times higher. The relatively small elasticities are consistent with the results shown graphically in Figs 3 and 4; even though the simulated catch rates vary substantially over time, the effect on the distribution of trips to the different bays is relatively slight.

**Table 5:** Estimates of elasticities, $\varepsilon_{j}^{T}$, for different bays, seasons and species.

	Galveston	Matagorda	San Antonio	Aransas	Corpus Christi	Upper Laguna Madre	Lower Laguna Madre
***Elasticities evaluated at λ = 4*.*96 (cost per mile = $0*.*5)***							
**Red Drum**							
High Season	0.23	0.14	0.18	0.18	0.21	0.14	0.25
Low Season	0.22	0.16	0.12	0.15	0.12	0.11	0.18
**Spotted Sea Trout**							
High Season	0.78	0.45	0.55	0.52	0.41	0.28	0.54
Low Season	0.67	0.45	0.51	0.58	0.49	0.31	0.57
***Elasticities evaluated at λ = 2*.*46 (cost per mile = $1)***							
**Red Drum**							
High Season	0.15	0.11	0.15	0.14	0.17	0.11	0.20
Low Season	0.16	0.13	0.11	0.11	0.09	0.08	0.14
**Spotted Sea Trout**							
High Season	0.52	0.36	0.46	0.40	0.34	0.22	0.44
Low Season	0.49	0.37	0.44	0.42	0.39	0.23	0.45
***Elasticities evaluated at λ = 1*.*23 (cost per mile = $2)***							
**Red Drum**							
High Season	0.12	0.10	0.14	0.12	0.16	0.09	0.18
Low Season	0.13	0.12	0.10	0.09	0.08	0.07	0.12
**Spotted Sea Trout**							
High Season	0.39	0.32	0.42	0.35	0.31	0.19	0.39
Low Season	0.40	0.33	0.40	0.34	0.34	0.20	0.40

## Discussion

Our linked model simulates the number of trips to seven major bays along the Texas coast (Fig 4). The decisions by anglers are affected by the distance to fishing locations and expected catch rates of red drum and spotted seatrout, which are simulated with a biological model. The results are imperfect due to the fact that the recreation demand model was estimated using data from elsewhere in the Gulf of Mexico. Nonetheless, the approach should provide qualitative indicators of how changes in fish stocks will affect recreation behavior. As seen in Fig 4, changing stocks are estimated to have consequences for the total number of trips taken by Texas anglers, but the distribution of trips across the seven bays does not change dramatically. The magnitudes of the changes observed are not surprising given our estimates of the elasticities (Table 5). At a base cost per mile of $1, the elasticities average only 0.13 for red drum trips and 0.40 for spotted sea trout: modest changes in stocks will not lead to dramatic changes in recreational fishing behavior. Fish abundances are also positively correlated among the bays; this reduces the change in the distribution of trips across the bays.

Bioeconomic models have been a part of fisheries economics for decades [34]. In recent years, models have added realism in both the biological component and the economic model of fisher behavior. Smith et al. [35] is one strong example. The authors study the implications of marine protected areas. Using a relatively simplistic population model, and location choice model similar to that employed here, the authors take into account the fact that commercial fishing chooses between multiple locations at which they can fish. This allows the authors to study how opportunity costs, e.g. employment in an alternative activity, can affect the consequences of a marine protected area. Johnston, Arlinghaus and Dieckmann [10] is another example. They use a more complex biological model, but a similar utility-theoretic economic model, to evaluate how angler diversity affects the optimal regulations of a recreational fishery. Neither of these papers, however, is calibrated to an empirical setting. Data is often a limiting factor, and the more complex the system becomes, the more difficult it is to obtain the appropriate data to calibrate such a model.

Despite our model’s empirical limitations, we believe it has some important utility. We show how an empirical model of stock dynamics might be linked to an economic model of recreational behavior to predict changes in recreational efforts in the coming season. Sampling for the fish monitoring is done in spring. As long as the CPUE in the monitoring data is a good indicator of actual fish abundance, the CPUE can be used for predicting how many anglers would fish and where they are likely to go during the upcoming fishing season. If we also collect data for fishing site characteristics, we could understand the changes in the behavior of anglers in response to improving the available amenities in fishing sites. Further studies are clearly needed, but by providing a framework for such analysis, our study is one of the first steps in understanding the changes in behaviors of marine recreational anglers. At the same time, we demonstrate how empirically relevant estimates can be obtained, even when the available data are less than ideal.

Our biological model focuses on stationary fluctuation in the abundance of fishes. It was possible to incorporate non-stationary changes in fish abundance (Fig 2C & 2D) by extrapolating. However, extrapolating polynomial functions is problematic, so we opted not to incorporate them. The abundance of red drum in general and spotted seatrout in some bays are currently increasing. This is often attributed to hatchery release effort [36] and/or reduced bycatch in shrimp fisheries. If we can make predictions of how the abundance of the two species change over time, we could incorporate them into the model. It will tell us how anglers would respond to such changes using the current model.

Our biological and angler’s decision models still have a room for improvement. For example, our current biological model does not include the increased mortality due to increased recreational fishing effort. The development of such a model is limited by the inability to separate fishing mortality and natural mortality. In addition, both species are potentially affected by bycatch mortality from commercial shrimp fisheries. Separating these causes of mortalities is challenging. We also do not clearly understand the natural density dependent feedback to the mortality of the two species. In order to incorporate the increased fishing effort into our model, it is important to tease out these different sources of mortality and how they change with the abundance of fish and fishing effort. Once these parameters are estimated, they can be incorporated into mode detailed population models, replacing VARMs.

One of the limitations of the angler’s decision model is that the empirical model captures the sensitivity of trip choice to distance, not to cost directly. Hence, our estimates accurately capture anglers’ sensitivity to the travel cost only correct if that cost is proportional to the distance traveled. We see that as the cost per mile declines, the estimated coefficient on travel cost increases and we predict that anglers will be substantially more responsive to changing biological conditions.

Finally, as pointed out previously, the discrepancy in the locations of the data sets is a limitation of the current study. In particular, the data for the anglers’ decision model were from west Florida, Alabama, and Mississippi whereas the data for the biological model were from Texas. Therefore, it needs to be interpreted carefully. If we can assume that anglers behave similarly in the eastern and western parts of the northern Gulf of Mexico, then, we can interpret the results directly. To some extent, behaviors almost certainly are different. The results, therefore, should be interpreted as indicative of the kind of responses that we might expect as fish stocks fluctuate over time, but not as precise predictions. Nonetheless, the model demonstrates the potential value of asking hypothetical questions by combining the information obtained from geographically or temporarily separated data sets. We find evidence that the total trips by anglers are likely to change in response to changes in the stocks of red drum and spotted seatrout and the empirically based model indicates the magnitude of changes that one might expect. This type of question can only be answered by developing an integrated model like the one we present here.

## Supporting information

S1 Table Catch per unit effort of red drum and spotted seatrout during spring and fall sampling. (XLSX)

S2 Table Ten top vector autoregressive state-space models for red drum based on BIC. (DOCX)

S3 Table Ten top vector autoregressive state-space models for spotted seatrout based on BIC. (DOCX)
