# Supplementary material for: A coupled recreational anglers’ decision and fish population dynamics model
Source: PLoS One. 2018 Oct 31;13(10):e0206537. doi: 10.1371/journal.pone.0206537 (PMC6209354; doi:10.1371/journal.pone.0206537)
Supplement: S2 Table — (DOCX) [file pone.0206537.s002.docx]

**Supporting information for Fujiwara et al. (2018) A coupled recreational anglers’ decision and fish population dynamics model. PLOS One.**

TABLE S2. Ten top vector autoregressive state-space models for red drum based on BIC. “x” indicates the parameter was included in the model. The full equations are shown in the main text (equations 1 and 2). ΔBIC is the delta BIC. The estimated parameters for the best model is shown in Table 1 of the main text.

| $b_{1,1}$ | $b_{2,1}$ | $b_{1,2}$ | $b_{2,2}$ | $w_{3,1}$ | $w_{4,1}$ | $w_{2,1}$ | $w_{5,1}$ | $w_{1,1}$ | $w_{3,2}$ | $w_{4,2}$ | $w_{2,2}$ | $w_{5,2}$ | $w_{1,2}$ | $\nu_{1}$ | $\nu_{2}$ | ΔBIC |
| --- | --- | --- | --- | --- | --- | --- | --- | --- | --- | --- | --- | --- | --- | --- | --- | --- |
|  | x |  | x | x | x |  |  | x | x |  | x |  | x | x | x | 0 |
|  | x |  | x | x | x |  |  | x | x | x |  |  | x | x | x | 0.22 |
|  | x |  | x | x |  | x |  |  | x |  | x |  | x | x | x | 1.15 |
|  | x |  | x | x |  | x |  |  | x | x |  |  | x | x | x | 1.38 |
|  | x |  | x | x |  | x |  | x | x |  | x |  | x | x | x | 1.44 |
|  | x |  | x | x | x |  |  |  | x |  | x |  | x | x | x | 1.62 |
|  | x |  | x | x |  | x |  | x | x | x |  |  | x | x | x | 1.67 |
|  | x |  | x | x | x |  |  |  | x | x |  |  | x | x | x | 1.85 |
| x | x |  | x | x | x |  |  | x | x |  | x |  | x | x | x | 2.01 |
| x | x |  | x | x |  | x |  |  | x |  | x |  | x | x | x | 2.03 |
